# Communities Setting the Direction for Their Right to Nutritious, Affordable Food: Co-Design of the Remote Food Security Project in Australian Indigenous Communities

**DOI:** 10.3390/ijerph20042936

**Published:** 2023-02-08

**Authors:** Megan Ferguson, Emma Tonkin, Julie Brimblecombe, Amanda Lee, Bronwyn Fredericks, Katherine Cullerton, Catherine L. Mah, Clare Brown, Emma McMahon, Mark D. Chatfield, Eddie Miles, Yvonne Cadet-James

**Affiliations:** 1 School of Public Health, The University of Queensland, Herston, QLD 4006, Australia; 2 Wellbeing and Preventable Chronic Disease Division, Menzies School of Health Research, Charles Darwin University, Casuarina, NT 0810, Australia; 3 Department of Nutrition, Dietetics and Food, Faculty of Medicine Nursing and Health Sciences, Monash University, Clayton, VIC 3168, Australia; 4 Office of the Pro-Vice Chancellor (Indigenous Engagement), The University of Queensland, St. Lucia, QLD 4006, Australia; 5 School of Health Administration, Dalhousie University, Halifax, NS B3H 4R2, Canada; 6 Apunipima Cape York Health Council, Bungalow, QLD 4870, Australia; 7 Indigenous Education and Research Centre, James Cook University, Bungalow, QLD 4870, Australia

**Keywords:** Aboriginal and Torres Strait Islander, first nations, food security, diet quality, co-design

## Abstract

Despite long histories of traditional food security, Indigenous peoples globally are disproportionately exposed to food insecurity. Addressing this imbalance must be a partnership led by Indigenous peoples in accordance with the UN Declaration of the Rights of Indigenous Peoples. We report the co-design process and resulting design of a food security research project in remote Australia and examine how the co-design process considered Indigenous peoples’ ways of knowing, being, and doing using the CREATE Tool. Informed by the Research for Impact Tool, together Aboriginal Community Controlled Health Organisation staff, Indigenous and non-Indigenous public health researchers designed the project from 2018–2019, over a series of workshops and through the establishment of research advisory groups. The resulting Remote Food Security Project includes two phases. Phase 1 determines the impact of a healthy food price discount strategy on the diet quality of women and children, and the experience of food (in)security in remote communities in Australia. In Phase 2, community members propose solutions to improve food security and develop a translation plan. Examination with the CREATE Tool showed that employing a co-design process guided by a best practice tool has resulted in a research design that responds to calls for food security in remote Indigenous communities in Australia. The design takes a strengths-based approach consistent with a human rights, social justice, and broader empowerment agenda. Trial registration: The trial included in Phase 1 of this project has been registered with Australian New Zealand Clinical Trials Registry: ACTRN12621000640808.

## 1. Introduction

Aboriginal and Torres Strait Islander peoples in Australia maintained food security for tens of thousands of years through their detailed knowledge of Country and their food systems [1,2], resulting in varied and high-quality diets [1,3]. Yet the cumulative impact of the atrocities inflicted upon Aboriginal and Torres Strait Islander peoples during colonisation, including the taking of land and resources, and social injustices, have left the population exposed to food insecurity, particularly people living in remote areas [4].

Food security is defined globally to “exist when all people, at all times, have physical, social and economic access to sufficient, safe and nutritious food that meets their dietary needs and food preferences for an active and healthy life” [5] (p. 28). It is a basic human right that people have a fair opportunity, now and in the future, to have autonomy over when and what they eat [6]. With nearly one in three people in the world not having physical and/or economic access to adequate food in 2021 [7], now more than ever, work must be done to address the causes of food insecurity globally to reach the 2030 development goal of a world with no hunger [7]. In Australia, 31% of Aboriginal and Torres Strait Islander peoples living in remote areas were reported to be food insecure, compared to 22% of all Aboriginal and Torres Strait Islander peoples and 4% of the wider population [4]. This is likely to be an under-estimate, with research suggesting it could be as high as 62% in remote communities in the Northern Territory (NT) [8]. As experienced globally [7], food insecurity in Australia has been exacerbated by the COVID-19 pandemic, which has exposed the pre-existing, systematic failures in ensuring food security in remote Aboriginal and Torres Strait Islander communities [9].

Improving food security with Indigenous peoples both in Australia and globally requires a participatory approach, in line with the human rights principle of participation [10]. This ensures Indigenous peoples shape the strategies, policies, and programmes promoting the realisation of their right to food [11]. When Indigenous peoples control decisions affecting them, their cultural determinants of health and wellbeing are protected. This occurs by the maintenance and strengthening of institutions, cultures, and traditions. It also occurs by promotion of development in accordance with their own needs, priorities, and goals [10,12]. As such, research aimed at improving food security in remote Australia must be led by Indigenous peoples. It must value and incorporate Aboriginal and Torres Strait Islander knowledge and understandings and be both decolonising and strengths-based [12]. A co-design development process, here defined as an ‘active collaboration between stakeholders in designing solutions to a prespecified problem’ [13](p. 2) is one way to help achieve this.

One tool available for informing a co-design process is the Lowitja Institute’s Research for Impact Tool (Research for Impact Tool) (Table 1) [14]. The Research for Impact Tool identifies and describes a process for planning research with Aboriginal and Torres Strait Islander peoples, with a focus on planning for, translating, and evaluating research impact. A complementary quality appraisal tool (The Aboriginal and Torres Strait Islander Quality Appraisal Tool) designed by the Centre of Research Excellence in Aboriginal Chronic Disease Knowledge Translation and Exchange (hereafter the ‘CREATE Tool’) (Table 1) can assist researchers in ensuring their research reflects Aboriginal and Torres Strait Islander peoples’ ways of knowing, being, and doing [15]. The developers encourage the use of the tool in planning research ‘to achieve appropriate, high quality and relevant research that benefits Aboriginal and Torres Strait Islander peoples’ [15]. Both tools emphasise the importance of Aboriginal and Torres Strait Islander leadership and partnership in carrying out research in the field, as well as the primary requirement for the research to benefit Aboriginal and Torres Strait Islander peoples.

The first item of the CREATE Tool reflects the tenet of decolonising research. It states that Aboriginal and Torres Strait Islander communities should choose if, when, and how to engage non-Indigenous researchers in research [16], set research priorities [12], and direct research approaches and methods. In Australia, Aboriginal Community Controlled Health Organisations (ACCHOs) are primary health care services initiated and operated by the local Aboriginal community. ACCHOs deliver holistic, comprehensive, and culturally appropriate health care to the community which controls it, through a locally elected Board of Management [17]. Being on the frontline of community healthcare, ACCHOs also set and communicate community identified health research priorities [18]. Therefore, these services in communities are supported by staff who advocate for the needs communities themselves define, and are key in facilitating connection between the community and health researchers [18]. The northern communities that Apunipima Cape York Health Council (Apunipima), the largest ACCHO in Queensland support, consider food insecurity to be a priority for health research and action. Thus, Apunipima released its Board-endorsed Food Security Position Statement in 2017 which calls for comprehensive cross-sectoral action to improve food security for remote Cape York communities [19].

In 2010, in a community-level food security project, Aboriginal peoples in remote North Queensland and the NT determined their own definition of food security, that being, “when the food of our ancestors is protected and always there for us and our children. It is also when we can easily access and afford the right non-traditional food for a collective healthy and active life. When we are food secure we can provide, share and fulfil our responsibilities, we can choose good food knowing how to make choices and how to prepare and use it” [20]. Causes of food insecurity in remote communities relate to all food security dimensions; agency, stability, sustainability, access, availability, and utilization [1,5,21,22]. Whilst some causes may be community specific, others such as high food prices (ranging from 31–60% higher than major urban centres [23,24,25]) are common [26]. In response, Apunipima led a project to focus on the economic access factors of food security. In 2015, they trialled a fruit and vegetable voucher reward system. Based on the findings of this study, they recommended that a trial of a subsidy on a range of healthy foods, delivered through a system such as a discount card, for women and children [27] who are particularly at risk of food insecurity [28], be conducted. Apunipima wrote this into a call for action [19].

This research directly responds to Apunipima’s call to action. Understanding that research must have Aboriginal and Torres Strait Islander leadership if it is to appropriately reflect Aboriginal and Torres Strait Islander ways of knowing, being, and doing [12], the research response was co-designed in partnership between ACCHO staff and national and international nutrition, food security, and policy academics. The team included both Aboriginal and Torres Strait Islander and non-Indigenous people.

In a long-term research program with ACCHOs which has resulted in genuine community benefit, strong credible leadership, including senior Aboriginal leadership, was one key success factor identified in developing trusting relationships with shared power [29]. Despite their value, there are few reports of the process used in co-designing research among Aboriginal and Torres Strait Islander communities [30], and Indigenous populations globally, where specifically a lack of tools, frameworks, and description of implementing these in practice, has been highlighted [31]. This paper therefore aims to address this gap, by documenting the process of co-design used, and outlining the resulting design for the Remote Food Security Project. In reporting this work, we describe the co-design process in relation to the Research for Impact Tool, and outline in the methods and results how the resulting project aligns with the CREATE Tool.

## 2. Materials and Methods

### 2.1. Framework

The Remote Food Security project co-design process was initiated in 2018–2019, in response to a request for action from remote Cape York Aboriginal communities, in the form of Apunipima’s call to action to address food insecurity. The co-design process undertaken can be described by linking to the Research for Impact Tool which outlines six steps for designing research [14]. This tool, published in 2016, was the most relevant guide at the time for planning our research to ensure it would have impact. Since the development of the Research for Impact Tool and our co-design process, the CREATE Tool has been published (2020) [15]. This tool adds consideration of research quality beyond impact. The output of this co-design process (the Remote Food Security Project) was assessed using the CREATE Tool. In demonstration of its practical application to our process, we identify in this paper where we have assessed elements of our process as meeting a CREATE Tool item. This identification informed our reflection of the use this tool to evaluate our co-design process. Table 1 maps our interpretation of the items of the CREATE Tool to the steps of the Research for Impact Tool. Our interpretation is that there is a high level of congruence between these tools. The Research for Impact Tool ensures a focus on early design aspects, and the CREATE Tool (with its focus on quality appraisal) provides more specific elaboration on how steps such as leadership might be articulated in research with Aboriginal and Torres Strait Islander communities. 

### 2.2. Co-Design Team

The early co-design team aimed for the research to have relevance and impact not only in the remote region of Cape York, but across Australia. It was considered essential that perspectives from diverse groups of Aboriginal and Torres Strait Islander people be represented throughout the research [12]. Central Australian Aboriginal Congress (Congress), the largest ACCHO in the NT, was identified as a natural partner. Congress recognises that food security is a major concern in Central Australia. It is a priority action area for their Board [18]. This extended partnership would also provide an opportunity for ACCHOs to learn together in co-leading research.

The co-design team comprised nutrition, population health, and research staff from Apunipima, Aboriginal and Torres Strait Islander public health researchers, and non-Indigenous public health researchers, with input from Congress’ research coordinator (CREATE Tool item 9). Therefore, the research team has and will benefit from leadership from Aboriginal and Torres Strait Islander health researchers, as well as ACCHO staff (CREATE Tool item 3). There was, and continues to be, a strong focus on two-way learning identified by the group (CREATE Tool item 14). Indigenous and non-Indigenous researchers have opportunities to learn from one another, and to work alongside ACCHO staff and community members in different contexts. Research capacity has been strengthened for ACCHO staff and community members through involvement in project planning. This will continue throughout project implementation (CREATE Tool item 13).

### 2.3. Co-Design Process

In line with the Research for Impact Tool step 1, we defined users of the research outcomes to be remote community members and the ACCHOs servicing them. Face-to-face and virtual meetings were conducted from September 2018 to March 2019. Academics presented ACCHO staff with an appraisal of the literature (step 2) that addressed the broad areas of approaches to deliver a direct-to-consumer food subsidy and define and disseminate solutions through the voice of those experiencing food insecurity. Based on this shared evidence, the ACCHO staff and academics determined that the following three key components would be acceptable and feasible for the project (step 3): implementation of a healthy food price discount strategy to test how price discounts impact the diet of mothers and children; capturing experiences through photos (photo voice) to develop a range of solutions to inform policy to improve food security; and finally, bringing this together to develop a community-led framework to improve food security (CREATE Tool item 9). ACCHO staff contributed community- and regional-level evidence to inform the design. Knowledge translation (step 5), being a key requirement of ACCHOs, was explicitly built into the design through the community-led framework. It is also incorporated throughout the research plan through dissemination of research findings with Community Advisory Groups, ACCHOs, and, when approved by these groups, the broader public (CREATE Tool item 11). Qualitative feedback from Community Advisory Groups and ACCHOs will contribute to an assessment of the research benefits, and benefits beyond the research (steps 4 and 6, and CREATE Tool item 12).

## 3. Results

### 3.1. Setting

The research was planned to be conducted across both the regions of Cape York in Queensland and Central Australia in the NT (CREATE Tool item 2). Classifications of remoteness used in Australia account for geographical distance from major cities, as well as accessibility to service centres and population size [32], with remote and very remote communities being many hours’ drive from major service centres. Apunipima services 11 remote communities in Cape York [33] and Congress services six remote communities in Central Australia [34]. The process for ascertaining support and interest from individual communities within the regions was intended to be individualised to adapt to the governance structures within each community. Project communication was planned through local ACCHO governance groups such as Health Action Teams, Health Boards, and/or other local governance groups such as a Child and Family Centre reference group. These groups would provide a formal letter of support if they were interested in supporting the project within their community after receiving information via a Participant Information Sheet and Project Story.

### 3.2. Governance and Agreements

The Remote Food Security Project is designed with multiple levels of governance, with Aboriginal and Torres Strait Islander community or team member representation at all levels (CREATE Tool item 4). The governance for this project reflects long-standing relationships amongst academics and organisations, with active cultivation of reciprocity, trust, and respect over many years. The Chief Investigator Group is comprised of senior and emerging Aboriginal academics (CREATE Tool item 3) and representatives from both ACCHOs, as well as public health, nutrition, and policy academics, and is responsible for overseeing the overall study conduct. The Chief Investigator Group oversees three Working Groups which are responsible for advising on the development of research processes. The implementation of research processes is led by the two ACCHOs, with the support of additional research content specialists, all of whom make up the Implementation Team. Dedicated project managers are the face of the project with communities, responsible for liaising with Community Advisory Groups. Community Advisory Groups formally approve and direct project implementation within their community, ensuring local community protocols are respected and followed (CREATE Tool item 5). It was planned that project managers will conduct data collection alongside local Aboriginal researchers, with the project having a strong focus on capacity development through the training of local researchers in research processes like recruitment, consent, and data collection (CREATE Tool item 13). Having Indigenous involvement at all levels of governance, from the Chief Investigator Group to the local implementation teams, will ensure the project is highly sensitive to and inclusive of different Aboriginal and Torres Strait Islander perspectives (CREATE Tool item 2). A Multi-Institutional Agreement for the project was negotiated between Apunipima and the participating research institutions, outlining each organisation’s role, contribution, and funding for the project, as well as background and resulting intellectual property ownership and use. A similar Subcontract agreement was negotiated between Congress and The University of Queensland (CREATE Tool items 6 and 7). It was planned that participants will be informed during the consent process that ownership of Aboriginal and Torres Strait Islander knowledge and cultural heritage is retained by participants and will be acknowledged in the research findings (CREATE Tool item 6), with all outputs from the work being jointly owned and approved by the ACCHOs, as well as the participating research institutions (CREATE Tool item 7).

### 3.3. Ethics

The Remote Food Security Project has approval from the Research Governance Committee of Apunipima Cape York Health Council, Central Australian Aboriginal Congress Board (CREATE Tool item 4), Central Australian Human Research Ethics Committee (CA-20-3701), and The University of Queensland Human Research Ethics Committee B (2020000636). The consent procedures involve participants consenting separately to being interviewed, having their study data accessed, and for their data to be securely held at the research institutions. The participants can choose to consent to some aspects but not others if desired. In this way each participant has control over how their data is managed, including knowing their right to have their data withdrawn at any time without prejudice (CREATE Tool item 8).

### 3.4. Research Design and Objectives

Having the Apunipima Call to Action as the primary motivation for the Remote Food Security Project (CREATE Tool item 1), and Congress’ identified research priorities, ensured that the project was designed with the purpose of having communities as beneficiaries of the research (CREATE Tool item 12). A vital aspect of working within a decolonising research paradigm is that participants must be involved in determining how the research findings are used [12], and research data must be used to support group aspirations [35]. These concepts are embedded in the Remote Food Security Project through the project having two phases. Based on the evidence needs identified for end users and the appraisal of existing evidence conducted during the co-design process, Phase 1 focuses on the impact of a healthy food price discount strategy and learning about food insecurity in community. Phase 2 builds on the knowledge shared and created in Phase 1 to focus on benefits to communities: how to support food security in remote communities through use of photo voice and development of a community-led framework to improve food security. The Remote Food Security Project objectives are therefore:

#### 3.4.1. Phase 1

(1) To evaluate the impact of price discounting on healthy foods on (a) diet quality for women and children and (b) affordability of a healthy diet.
(2) To understand community members’ experiences of food insecurity.

#### 3.4.2. Phase 2

(1) To determine proposed solutions to improve food security through learning from community members.
(2) To develop a community framework and knowledge sharing/translation plan for improving food security.

### 3.5. Project Design

#### 3.5.1. Phase 1—Testing Phase

In Phase 1, the healthy food price discount strategy was designed to be assessed in terms of its impact on the diet quality of mothers and children and affordability of a healthy diet (CREATE Tool item 1). Qualitative interviews were designed to be used to understand, in the words of community members, the food insecurity story in remote communities. All findings were planned to be presented to Community Advisory Groups and local Aboriginal researchers for advice regarding interpretation (CREATE Tool item 14).

##### Heathy Food Price Discount Strategy

The co-design team determined that the best approach to explore the value of a healthy food price discount strategy to support food security in remote communities was a controlled before-and-after design. The design included eligible participants having access to a discount card that unlocks price discounts on healthy food purchases in the community store. The primary outcome is the difference in the change from baseline in diet quality between participants in strategy and control communities. We also planned to assess how the discounted prices impacted affordability. Secondary outcomes include food security and anaemia.

In line with Apunipima’s targeted call, all Aboriginal and/or Torres Strait Islander pregnant and breastfeeding women, and parents/primary carers of children aged six months to five years who identify as a resident and plan to reside in the community for the trial period were eligible. Timelines for recruitment were to be directed by Community Advisory Groups to best avoid times of community business, therefore respecting local community protocols (CREATE Tool item 5) and upholding the right for communities themselves to have control over data collection (CREATE Tool item 8). It was planned that one discount card identity be allocated per family, with up to three cards linked to the identity as requested by participants to meet their family needs. The design included three capped fortnightly levels of discount depending on the number of eligible household members: $80, $100, or $120 each fortnight for one, two, or three or more eligible people, respectively. It was estimated that $120 would promote an affordable diet in line with the accepted benchmark of food spend being 30% of household income for a family of four [36].

We planned for the trial to be conducted in two strategy and two control communities in each region, totalling eight communities. Inclusion criteria included: (i) previous or expressed commitment from council, Health Boards, Health Action Teams, or other local governing bodies to support food and nutrition projects, (ii) existence of strong community groups, and (iii) for strategy communities, that the store operates a point-of-sale system to support the delivery of the discount strategy. We anticipated to have 240 participants in each of the intervention and control groups, 480 in total after non-consent and drop-out. This sample size enables 80% power to detect an improvement of four (SD = 12) points in diet quality between intervention and control communities, assuming high uptake of the strategy [37]. During our co-design meetings and based on our collective experience, three months was considered the minimum time required for participants to make changes to their purchasing [27]; therefore, the discount period was determined to be three to six months in each strategy community. It was planned that Community Advisory Groups participate in selecting foods targeted for discounting in their community store based on the five food groups and healthy fat group of the Australian Dietary Guidelines [38], and therefore have control over this critical aspect of strategy implementation (CREATE Tool item 8). The design included a discount of 30% applied to all approved healthy foods and drinks in each community, as this approximates the price gap between remote stores and supermarkets in Queensland [23,39].

The implementation of the healthy food discount strategy was planned to be monitored through fortnightly review of store data of discount card purchases and monthly visits to communities throughout the strategy period by the ACCHO project managers. A survey assessing participant satisfaction with the discount card and strategy was planned to be conducted with participants in strategy communities at follow-up to gain insight into the success of the strategy from the participant perspective, and any areas where improvement could be made. This is key for informing the sustainability of any potential future discount programs introduced through translation of this work into policy (CREATE Tool item 11).

##### Data Collection

The primary outcome of diet quality was planned to be measured using a 32-item food frequency questionnaire which was developed with and for remote Aboriginal populations [40]. A Dietary Guideline Index score (score 0–100) [41], validated in a similar population [42], measuring the degree of adherence of the diet to the Australian Dietary Guidelines was planned to be calculated. Optional feedback to participants about their diet based on their score at follow up data collection was included in the design. The affordability of a healthy diet was planned to be measured at baseline and follow up [36].

A modified version of the United States Department of Agriculture 18-item Household Food Security Scale Module [43], which has been validated and used in research in Australia [44,45], was planned to be used to measure the prevalence of food insecurity. Haemoglobin data routinely collected at health clinics for two months prior to baseline up to strategy end were planned to be used to determine anaemia incidence and prevalence and will be compared between strategy and control communities at baseline and follow-up.

Contextual and supporting data includes demographic and household data for all participants, diet cost [36], minimum meal frequency data [46], an iron score and discount card purchasing data. All data collection tools were planned to be tested initially with ACCHO staff, and where possible, with local community members prior to use (CREATE Tool item 8).

##### Data Analysis

The difference in Dietary Guideline Index scores and food security status between strategy and control communities will be reported. Dietary intake and quality, food security status, anaemia incidence and prevalence, and discount card use will also be described. Diet cost and affordability data will be analysed using existing purpose-built data spreadsheets [36].

##### Qualitative Interviews

The design included the conduct of qualitative interviews with a sub-sample of 10–15 individual participants in each strategy community at baseline. The interviews were planned to be informed by an interview guide based on the literature including a study with urban Aboriginal and Torres Strait Islander people [47], exploring community members’ experiences of food insecurity. Data was planned to be managed in NVivo and thematically analysed, including by Aboriginal and Torres Strait Islander team members, and the findings interpreted by the wider research group as described above.

#### 3.5.2. Phase 2—Translation Phase

##### Design

The translational phase was designed to be informed by the outcomes of phase 1. We planned to use a qualitative methodology to develop a community-led framework and translation plan, with the goal of translating the research findings into sustainable changes in policy and practice (CREATE Tool item 11). This participatory design privileges the voices of community members, and therefore is inherently strengths-based (CREATE Tool item 10). Here, we outline early and broad plans, but it is intended that the process leading to the development of these outcomes will be informed by Community Advisory Groups, and therefore be community led.

First, photo voice methodology was planned to be employed to give a voice to remote community members’ lived experience of, and proposed solutions to, improving food security. Second, results from Phase 1 and the photo voice study, as well as evidence from recent food security events and inquiries, was planned to be presented to leaders within each participating community at community prioritisation workshops, with the aim to reach agreement on community-supported priorities and/or solutions to improve food security. Third, the community prioritisation results were planned to be considered at a knowledge exchange workshop facilitating the development of the community-led framework and translation plan (CREATE Tool item 11). To increase the translation of evidence into policy, concurrent to the aforementioned activities, we planned to test messaging using different frames to determine which is the most effective frame to address the key issues and solutions. This will ensure public receptiveness of the policy message and maximise traction of advocacy efforts to primarily non-Indigenous policymakers (CREATE Tool item 11).

##### Participants

It was planned that the participants in Phase 1 strategy communities be recruited for photo voice methodology. Community Advisory Groups can advise on who within each community should attend the community prioritisation workshops (CREATE Tool item 5). The knowledge exchange was planned to include up to 40 stakeholders, led by Community Advisory Groups with two representatives from each community (CREATE Tool item 8), as well as researchers, and policymakers. The design of the frame testing, to be conducted in the Gold Coast, Queensland, and Melbourne, Victoria, includes recruitment of members of the public aged over 18 years who speak English: at least 40 in intercept interviews and 20 in focus groups. A Qualtrics online panel was planned to be used to recruit 2000 Australian citizens in urban and regional locations who speak English and are aged over 18 years for the experimental framing study, with stratified sampling by age, gender, rural/urban location, school level, and voting preference to ensure the sample is representative of the wider Australian population.

### 3.6. Data Collection and Analysis

#### Photo Voice

Informed by Witnesses to Hunger [48], the co-design team aims to give greater voice to remote community members’ lived experience of food insecurity. The process was designed to be conducted in strategy communities, determined in conjunction with each Community Advisory Group and participants (CREATE Tool item 8). It was planned that participants have the opportunity to learn basic photography skills and about legal issues relating to privacy and copyright, representing capacity building for the community members participating (CREATE Tool item 13). It was planned that the key learnings from Phase 1 be shared, and the participants then take photos over an agreed timeframe to share their experience of and solutions to food insecurity. Information on theme generation is then shared, followed by participants collectively discussing the photos and arranging them into themes. The photos of most significance to participants are then discussed [49]. The researchers and participants will work together to determine how the data will be shared for the community prioritisation workshop in their community, and the knowledge exchange. In this way, participants have control over the data collection and interpretation (CREATE Tool item 8) and are assisted in presenting their stories of food security and insecurity and put forward policy solutions they feel are needed. This strengths-based approach inherently moves beyond practices of non-Indigenous researchers ‘reporting on problems in’ Aboriginal populations (CREATE Tool item 10).

### 3.7. Community Prioritisation Workshops

The community prioritisation workshops were designed to be informed by consensus building and deliberative democratic approaches [50,51,52]. The aim of the workshops is to achieve consideration of evidence and collective decision-making by community leaders, and agreement about community-supported priorities to improve food security to be shared at the knowledge exchange. The meeting procedure was planned to be negotiated with Community Advisory Groups (CREATE Tool item 5) and include the presentation of evidence, facilitated discussion, and finally prioritisation of the priorities and/or solutions to improve food security using the Ripple Tool [53]. The expected outcome is that representatives from each community will provide a summary of the discussion and final prioritisation outcomes for their community at the knowledge exchange.

### 3.8. Knowledge Exchange

Finally, we planned to bring together representatives from all strategy and control communities to conduct a knowledge exchange to develop a community-led framework to improve household food security in remote communities (CREATE Tool item 11). This was planned to include mapping of evidence from Phase 1, and the preceding parts of Phase 2 against existing food security determinant frameworks [54]. A translation plan will be developed, informed by a frame analysis.

### 3.9. Frame Analysis

We planned to develop and test with the public a range of frames, informed by the photo voice methodology and the community prioritisation outcomes. We planned to conduct intercept surveys in public spaces which collect age, gender, level of education, voting intention, and quantitative level of agreement and qualitative responses to different policy actions designed to improve food security among Aboriginal and Torres Strait Islander peoples. Descriptive analysis was planned to be conducted with demographic and level of agreement data, and a framing analysis will be undertaken with the qualitative data. Several pilot messages using the key frames from the intercept surveys will be developed. Through focus groups, we planned to then explore how understandable these pilot messages are and if they provoke thoughts or feelings towards the policy actions. Qualitative data will be managed in NVivo and thematically analysed. The messages will be further refined based on the focus group feedback. We then expect to conduct an experimental framing study using the three most effective messages from the focus groups plus a control. This study will determine which message framing will have greatest likelihood of increasing the acceptability of the proposed policy solution. Participants are planned to be stratified into four nationally representative groups which will receive the same policy solution framed in different ways. A short survey on level of support for the policy was planned, asking participants how they feel about the policy, and whether they think the policy will be effective.

## 4. Discussion

Through the co-design process employed in its design, the Remote Food Security Project takes a strengths-based approach that fits within a human rights, social justice, and empowerment agenda, therefore responding to calls from ACCHOs to support food security in remote Aboriginal and Torres Strait Islander communities in Australia. The result of having the co-design process underpinned by the Research for Impact Tool is a project that delivers on a solution proposed in Apunipima’s call to action and aligned with Congress’ research priorities, uses participatory methods, and has a strong translation focus. A challenge found in applying this best practice tool was in negotiating details with community members within tight grant timelines, and in being mindful of taking time and setting expectations in the community without guaranteed funding. However, in defining the evidence needs of the users, it was clear that we were responding to Apunipima’s call to action. We also ensured that the design which was participatory in nature allowed for community direction; and this has been shown, for example, by who governs and directs the project locally.

As identified, there was significant overlap between these two complementary tools, which was further highlighted when the CREATE Tool was applied to our co-design process in line with the Research for Impact Tool steps. We found the Research for Impact Tool useful in considering our broad approach that focused the design development on the users’ needs (steps 1–3) and in aiming to deliver genuine benefit to communities (steps 4–6). The CREATE Tool, which similarly focuses on these issues, allowed us to check on the steps we were planning in the research process. All tool items have been addressed in the design of the Remote Food Security Project. However, whilst our agreements with ACCHOs are legally binding, our agreements with Community Advisory Groups are in line with processes conducted in the regions and are governed by our ethics applications and are not legally binding (CREATE Tool item 6). We believe these two tools, especially when used together, support co-design processes that genuinely partner with Aboriginal and Torres Strait Islander communities and researchers for community benefit. Since our application of these tools, although not informed by a research lens, a review of optimal approaches to co-design in health care policy, practice, and service provision with Aboriginal and Torres Strait Islander people has been published [55]. The six themes of co-design identified in the review are reflected in the steps and questions of the tools we applied which are specific to the research setting. The review offers further detailed and practical sub-themes which would assist in operationalising these broad themes. Partnerships between peak health organisations and academics, especially when guided by best practice tools such as the Research for Impact Tool and the CREATE Tool, can strengthen research to deliver community benefit. This approach, undertaken in remote Australia, has application to other contexts nationally in considering the creation of evidence with groups impacted themselves, to inform policy decisions in line with the UN Declaration of the Rights of Indigenous Peoples.

The Remote Food Security Project directly responds to a call to action from Apunipima to test a direct-to-consumer food subsidy scheme to address financial barriers and increase affordability and access to healthy food and drink in remote areas. The price discount directly addresses the high cost of food in remote communities. Following testing of the price discount, the project will work with Community Advisory Groups and participants to collect local evidence that is expected to address other determinants of food security in Cape York and Central Australia. Together these forms of evidence will provide community members and the ACCHOs servicing them with a collective understanding of the many factors influencing food insecurity, and a picture of the solutions for policymakers, in community members’ own words. Our message testing with the public will help ensure these policy messages are informed by what is considered broadly acceptable, and therefore have the best opportunity to gain traction in policy settings.

### Strengths and Limitations

This approach undertaken in remote Australia, has informed the design of a project that will ensure the creation of evidence with the groups impacted themselves, to inform policy decisions in line with the UN Declaration of the Rights of Indigenous Peoples. Whilst we have found the Research for Impact and CREATE Tools useful in planning and assessing our co-design process, we acknowledge that although these may be useful to Indigenous peoples and academics working together elsewhere in the world, these tools were developed by and for, Australian Aboriginal and Torres Strait Islander peoples.

As per the Apunipima call to action, we have targeted women and children within the context of the family as a priority for inter-generational change, but ultimately food insecurity must be addressed for all. As the casual factors for food insecurity are common, the proposed solutions are potentially of benefit to all remote communities. Any research that builds on this study or policy influence resulting from this study is likely to be applied to, and therefore impact on, remote communities broadly.

## 5. Conclusions

This work demonstrates that co-design of research to address community-identified needs, informed by best practice tools, is feasible, and results in project design which highly aligns with general ethical frameworks to support the human rights of Indigenous peoples.

## Figures and Tables

**Table 1:** The CREATE Tool items [15] (p. 8) mapped to the Research for Impact Tool steps [14] (p. 8).

Research for Impact Tool Steps	CREATE Tool Items
**1.** Define the users and critically assess their evidence needs	**1.** Did the research respond to a need or priority determined by the community?
**2.** Appraise the existing evidence	
**3.** Select and implement an appropriate research type and design	**9.** Was the research guided by an Indigenous research paradigm? **10.** Does the research take a strengths-based approach, acknowledging and moving beyond practices that have harmed Aboriginal and Torres Strait peoples in the past?
**4.** Assess the project level benefits and costs **6.** Assess the benefits beyond the project versus costs	**12.** Did the research benefit the participants and Aboriginal and Torres Strait Islander communities?
**5.** Translate the new knowledge to influence decisions and actions beyond the project	**11.** Did the researchers plan to and translate the findings into sustainable changes in policy and/or practice?
Aboriginal and Torres Strait Islander leadership, economic evaluation and knowledge translation expertise, partnership, learning by doing, and capacity enhancement within each step	**2.** Was community consultation and engagement appropriately inclusive? **3.** Did the research have Aboriginal and Torres Strait Islander research leadership? **4.** Did the research have Aboriginal and Torres Strait Islander governance? **5.** Were local community protocols respected and followed? **6.** Did the researchers negotiate agreements in regards to rights of access to Aboriginal and Torres Strait Islander peoples’ *existing* intellectual and cultural property? **7.** Did the researchers negotiate agreements to protect Aboriginal and Torres Strait Islander peoples’ ownership of intellectual and cultural property *created* through the research? **8.** Did Aboriginal and Torres Strait Islander peoples and communities have control over the collection and management of research materials? **13.** Did the research demonstrate capacity strengthening for Aboriginal and Torres Strait Islander individuals? **14.** Did everyone involved in the research have opportunities to learn from each other?

## Data Availability

No new data were created or analysed in this study. Data sharing is not applicable to this article.

## References

[1] Lee A., Ride K. (2018). Review of nutrition among Aboriginal and Torres Strait Islander people. Aust. Indig. Health Bull..

[2] Berndt R.M., Berndt C.H. (1964). The World of the First Australians. Aboriginal Traditional Life: Past and Present.

[3] O’Dea K. (1991). Traditional diet and food preferences of Australian aboriginal hunter-gatherers. Philos. Trans. R. Soc. Lond. Ser. B Biol. Sci..

[4] Australian Bureau of Statistics (2015). Australian Aboriginal and Torres Strait Islander Health Survey: Nutrition Results—Food and Nutrients, 2012–2013.

[5] HLPE (2020). Food Security and nutrition: Building a Global Narrative towards 2030.

[6] Eide W.B., Kracht U. (2005). Food and Human Rights in Development: Legal and Institutional Dimensions and Selected Topics: Volume 1.

[7] FAO, IFAD, UNICEF, WFP, WHO (2022). The State of Food Security and Nutrition in the World 2022.

[8] Brimblecombe J., Ferguson M., Barzi F., Brown C., Ball K. (2018). Mediators and moderators of nutrition intervention effects in remote Indigenous Australia. Br. J. Nutr..

[9] Fredericks B., Bradfield A. (2021). Indigenous Australians and COVID-19: Highlighting ongoing food security issues. Int. J. Home Econ..

[10] UN General Assembly United Nations Declaration on the Rights of Indigenous Peoples: Resolution/Adopted by the General Assembly. A/RES/61/295. https://www.refworld.org/docid/471355a82.html.

[11] FAO, Secretariat of the Permanent Forum of Indigenous People (2008). The Right to Food and Indigenous Peoples: Joint Brief.

[12] Harfield S., Pearson O., Morey K., Kite E., Canuto K., Glover K., Gomersall J.S., Carter D., Davy C., Aromataris E. (2020). Assessing the quality of health research from an Indigenous perspective: The Aboriginal and Torres Strait Islander quality appraisal tool. BMC Med. Res. Methodol..

[13] Vargas C., Whelan J., Brimblecombe J., Allender S. (2022). Co-creation, co-design, co-production for public health—A perspective on definitions and distinctions. Public Health Res. Pract..

[14] Tsey K., Lawson K., Kinchin I., Bainbridge R., McCalman J., Watkin F., Cadet-James Y., Rossetto A. (2016). Evaluating Research Impact: The Development of a Research for Impact Tool. Front. Public Health.

[15] Harfield S., Pearson O., Morey K., Kite E., Glover K., Canuto K., Gomersall K.S., Carter J., Davy D., Aromataris C. (2018). The Aboriginal and Torres Strait Islander Quality Appraisal Tool: Companion Document.

[16] Smith L.T. (2016). Decolonizing Methodologies: Research and Indigenous Peoples.

[17] NACCHO (2021). Aboriginal Community Controlled Health Organisations (ACCHOs) Canberra, ACT: National Aboriginal Community Controlled Health Organisation. https://www.naccho.org.au/acchos.

[18] Central Australian Aboriginal Congress (2019). Congress Research Strategy 2019–2023. https://www.caac.org.au/uploads/pdfs/Congress-Research-Strategy-2019-2023.pdf.

[19] Apunipima Cape York Health Council (2020). Position Statement: Food Security for Cape York Cairns: Apunipima. https://www.apunipima.org.au/wp-content/uploads/2020/02/positionstatement_foodsecuritycapeyork_final.pdf.

[20] Menzies School of Health Research (2016). Developing a Good Food System in your Community.

[21] Bryce S., Scales I., Herron L.-M., Wigginton B., Lewis M., Lee A., Ngaanyatjarra Pitjantjatjara Yankunytjatjara Npy Women’s Council (2020). Maitjara Wangkanyi: Insights from an Ethnographic Study of Food Practices of Households in Remote Australian Aboriginal Communities. Int. J. Environ. Res. Public Health.

[22] Brimblecombe J., Maypilama E., Colles S., Scarlett M., Dhurrkay J.G., Ritchie J., O’Dea K. (2014). Factors Influencing Food Choice in an Australian Aboriginal Community. Qual. Health Res..

[23] Lee A., Patay D., Herron L.-M., Parnell Harrison E., Lewis M. (2021). Affordability of current, and healthy, more equitable, sustainable diets by area of socioeconomic disadvantage and remoteness in Queensland: Insights into food choice. Int. J. Equity Health..

[24] Ferguson M., O’Dea K., Chatfield M., Moodie M., Altman J., Brimblecombe J. (2016). The comparative cost of food and beverages at remote Indigenous communities, Northern Territory, Australia. Aust. N. Z. J. Public Health.

[25] Northern Territory Government (2022). 2021 NT Market Basket Survey: Summary Report Northern Territory, Australia: Northern Territory Government. https://data.nt.gov.au/dataset/nt-market-basket-survey-2021/resource/3a02354a-b8ec-4be4-94dd-ddbd2c714c82.

[26] FAO, IFAD, UNICEF, WFP, WHO (2021). The State of Food Security and Nutrition in the World 2021. Transforming Food Systems for Food Security, Improved Nutrition And Affordable Healthy Diets for All.

[27] Brown C., Laws C., Leonard D., Campbell S., Merone L., Hammond M., Thompson K., Canuto K., Brimblecombe J. (2019). Healthy Choice Rewards: A Feasibility Trial of Incentives to Influence Consumer Food Choices in a Remote Australian Aboriginal Community. Int. J. Environ. Res. Public Health.

[28] Department of Health (2018). Healthy Under 5 Kids Program Growth and Nutrition NT Annual Report 2018 v1.0.1..

[29] Sherriff S.L., Miller H., Tong A., Williamson A., Muthayya S., Redman S., Bailey S., Eades S., Haynes A. (2019). Building trust and sharing power for co-creation in Aboriginal health research: A stakeholder interview study. Evid. Policy..

[30] Quigley R., Russell S.G., Sagigi B.R., Miller G., Strivens E. (2021). Community involvement to maximise research success in Torres Strait Islander populations: More than just ticking the boxes. Rural Remote Health.

[31] King P.T., Cormack D., Edwards R., Harris R., Paine S.-J. (2022). Co-design for indigenous and other children and young people from priority social groups: A systematic review. SSM—Popul. Health.

[32] Australian Bureau of Statistics (2013). 4727.0.55.002—Australian Aboriginal and Torres Strait Islander Health Survey: Users’ Guide, 2012–2013.

[33] Apunipima Cape York Health Council About 2022. https://www.apunipima.org.au/#:~:text=Apunipima%20Cape%20York%20Health%20Council,to%2011%20Cape%20York%20communities.

[34] Central Australian Aboriginal Congress About Us 2022. https://www.caac.org.au/about-us/.

[35] Walker P.O., Bretherton D., Law S.F. (2015). Indigenous Paradigm Research. Methodologies in Peace Psychology: Peace Research by Peaceful Means.

[36] Lee A., Lewis M. (2018). Testing the Price of Healthy and Current Diets in Remote Aboriginal Communities to Improve Food Security: Development of the Aboriginal and Torres Strait Islander Healthy Diets ASAP (Australian Standardised Affordability and Pricing) Methods. Int. J. Environ. Res Public Health.

[37] Grijalva-Eternod C.S., Jelle M., Haghparast-Bidgoli H., Colbourn T., Golden K., King S., Cox L.C., Morrsion J., Skordis-Worrall J., Fottrell E. (2018). A cash-based intervention and the risk of acute malnutrition in children aged 6–59 months living in internally displaced persons camps in Mogadishu, Somalia: A non-randomised cluster trial. PLoS Med..

[38] National Health and Medical Research Council (2013). Australian Dietary Guidelines.

[39] Queensland Health (2016). 2014 Healthy Food Access Basket Survey Brisbane: Queensland Government. https://www.health.qld.gov.au/research-reports/reports/public-health/food-nutrition/access/overview.

[40] Rohit A., Brimblecombe J., O’Dea K., Tonkin E., Maypilama L., Maple-Brown L. (2018). Development of a short-item diet quality questionnaire for Indigenous mothers and their young children: The Menzies remote short-item dietary assessment tool. Aust. J. Rural Health.

[41] Hendrie G.A., Smith E.V., Golley R.K. (2014). The reliability and relative validity of a diet index score for 4-11-year-old children derived from a parent-reported short food survey. Public Health Nutr..

[42] Tonkin E., Kennedy D., Golley R., Byrne R., Rohit A., Kearns T., Hanieh S., Biggs B.-A., Brimblecombe J. (2018). The Relative Validity of the Menzies Remote Short-Item Dietary Assessment Tool (MRSDAT) in Aboriginal Australian Children Aged 6–36 Months. Nutrients.

[43] Bickel G., Nord M., Price C., Hamilton W., Cook J. (2000). Guide to Measuring Household Food Security, Revised 2000.

[44] Ramsey R., Giskes K., Turrell G., Gallegos D. (2011). Food insecurity among Australian children: Potential determinants, health and developmental consequences. J. Child Health Care.

[45] McCarthy L. (2017). Household Food Security and Child Health Outcomes in Families with Children Aged 6 Months to 4 Years Residing in Darwin and Palmerston, Northern Territory, Australia.

[46] World Health Organisation (2010). Indicators for Assessing Infant and Young Child Feeding Practices Part 2: Measurement.

[47] McCarthy L., Chang A., Brimblecombe J. (2018). Food Security Experiences of Aboriginal and Torres Strait Islander Families with Young Children in An Urban Setting: Influencing Factors and Coping Strategies. Int. J. Environ. Res. Public Health.

[48] Chilton M., Rabinowich J., Council C., Breaux J. (2009). Witnesses to hunger: Participation through photovoice to ensure the right to food. Health Hum. Rights.

[49] Adams K., Burns C., Liebzeit A., Ryschka J., Thorpe S., Browne J. (2012). Use of participatory research and photo-voice to support urban Aboriginal healthy eating. Health Soc. Care Community.

[50] Innes J.E. (2004). Consensus Building: Clarifications for the Critics. Plan. Theory.

[51] Street J., Duszynski K., Krawczyk S., Braunack-Mayer A. (2014). The use of citizens’ juries in health policy decision-making: A systematic review. Soc. Sci. Med..

[52] Tonkin E., Wilson A.M., Coveney J., Meyer S.B., Henderson J., McCullum D., Webb T., Ward P.R. (2019). Consumers respond to a model for (re)building consumer trust in the food system. Food Control..

[53] Brimblecombe J., van den Boogaard C., Ritchie J., Bailie R., Coveney J., Liberato S. (2014). From targets to ripples: Tracing the process of developing a community capacity building appraisal tool with remote Australian indigenous communities to tackle food security. BMC Public Health.

[54] Brimblecombe J., Bailie R., Boogaard C.V.D., Wood B., Liberato S., Ferguson M., Coveney J., Jaenke R., Ritchie J. (2017). Feasibility of a novel participatory multi-sector continuous improvement approach to enhance food security in remote Indigenous Australian communities. SSM Popul. Health.

[55] Butler T., Gall A., Garvey G., Ngampromwongse K., Hector D., Turnbull S., Lucas K., Nehill C., Boltong A., Keefe D. (2022). A Comprehensive Review of Optimal Approaches to Co-Design in Health with First Nations Australians. Int. J. Environ. Res. Public Health.

